# Dose Escalation in Neoadjuvant Chemoradiotherapy for Rectal Cancer: Short-Term Efficacy and Toxicity of VMAT–SIB vs. 3D-CRT

**DOI:** 10.3390/medicina61030483

**Published:** 2025-03-11

**Authors:** Suzana Stojanovic-Rundic, Mladen Marinkovic, Aleksandra Stanojevic, Dusica Gavrilovic, Radmila Jankovic, Natasa Maksimovic, Aleksandar Tomasevic, Predrag Petrasinovic, Sandra Radenkovic, Milena Cavic

**Affiliations:** 1 Clinic for Radiation Oncology, Institute for Oncology and Radiology of Serbia, 11000 Belgrade, Serbia; stojanovics@ncrc.ac.rs (S.S.-R.); alektom@gmail.com (A.T.); ppetrasinovic@yahoo.com (P.P.); radenkovics6@gmail.com (S.R.); 2 Faculty of Medicine, University of Belgrade, 11000 Belgrade, Serbia; 3 Department of Experimental Oncology, Institute for Oncology and Radiology of Serbia, 11000 Belgrade, Serbia; astefanovic496@gmail.com (A.S.); jankovicr@ncrc.ac.rs (R.J.); milena.cavic@ncrc.ac.rs (M.C.); 4 Data Center, Institute for Oncology and Radiology of Serbia, 11000 Belgrade, Serbia; duca.gavrilovic@gmail.com; 5 Institute of Epidemiology, University of Belgrade, 11000 Belgrade, Serbia; natamax07@gmail.com

**Keywords:** locally advanced rectal cancer, neoadjuvant chemoradiotherapy, dose escalation, radiotherapy techniques, simultaneous integrated boost, sphincter preservation

## Abstract

*Background and Objectives*: The standard treatment for locally advanced rectal cancer (LARC) includes neoadjuvant chemoradiotherapy (nCRT), followed by surgery with or without adjuvant chemotherapy (CT). This study evaluated the efficacy and safety of dose-escalated radiotherapy (RT) using the volumetric modulated arc therapy–simultaneous integrated boost (VMAT–SIB) technique in patients with LARC compared to 3D conformal radiotherapy (3D-CRT). *Materials and Methods*: This study prospectively enrolled 75 patients with LARC. All patients received nCRT using VMAT–SIB, delivering a tumor dose (TD) of 54 Gy in 25 fractions, with concomitant CT following the 5-fluorouracil and leucovorin (5-FU–LV) protocol. To compare the treatment outcomes and toxicity associated with the increased RT dose, a retrospective cohort of 62 patients treated with the 3D-CRT technique was analyzed. The 3D-CRT group received a TD of 50.4 Gy in 28 fractions with the same CT. Outcomes, including pathological complete response (pCR), tumor regression grade (TRG), and sphincter preservation rates, were compared. *Results*: Among operated patients, the group treated with VMAT–SIB demonstrated improved rates of pCR (20.6% vs. 8.9%), with a statistically significant trend (*p* = 0.06). Sphincter-preserving surgeries were performed in 49 out of 63 operated patients (77.8%) in the VMAT–SIB group, compared to 35 out of 56 (62.5%) in the 3D-CRT group. Analysis of the definitive postoperative stage revealed a significantly higher prevalence of lower T categories (T0–2) (*p* < 0.01), negative N status (*p* < 0.05), and lower stages (I + II) (*p* < 0.05) in patients treated with the intensified RT approach. However, no significant differences in acute toxicity were observed. *Conclusions*: The implementation of intensified treatment with a higher dose using the VMAT–SIB technique demonstrated significant benefits in downsizing and downstaging compared to the standard treatment approach. These findings support its integration into clinical practice. However, further prospective, multi-center studies are needed to validate these results and assess long-term outcomes.

## 1. Introduction

Colorectal cancer ranked third in incidence and second in mortality among malignant diseases worldwide in 2022 [1]. Rectal cancer accounts for approximately 35% of all colorectal cancers. The standard treatment for locally advanced rectal cancer (LARC) includes neoadjuvant chemoradiotherapy (nCRT), followed by surgery with or without adjuvant chemotherapy (CT) [2].

Pathological complete regression (pCR) after nCRT is achieved in 10–30% of patients and is associated with better disease control and longer survival [3]. To achieve a higher rate of complete response (CR) to neoadjuvant therapy, research has focused on prolonging the interval between neoadjuvant treatment completion and surgery, increasing radiotherapy (RT) doses, and modifying the type and regimen of CT administration [4,5,6,7,8,9,10]. Advances in RT techniques have enabled dose escalation to the area of macroscopically visible disease, while better sparing surrounding organs at risk. The introduction of 3D conformal radiation therapy (3D-CRT) allowed for better adaptation (conformation) of the number and shape of radiation fields to the target volume. However, the anatomical proximity of the bladder and small intestine limited their maximal sparing. Simultaneous dose escalation within the tumor volume is achieved using intensity-modulated radiotherapy (IMRT) or volumetric modulated arc therapy (VMAT) techniques. This approach shortens the duration of nCRT compared to the conventional method, which involves dose escalation in a second phase of radiotherapy (sequential dose escalation).

Studies have demonstrated that dose-escalated RT can increase pCR rates and improve local disease control [11]. For example, Yang et al. reported a study using VMAT with a daily fraction dose of 2.35 Gy to a total tumor dose (TD) of 58.75 Gy in patients with distally located LARC, achieving 32% pCR, 60% sphincter preservation, and negative surgical margins in all patients [8]. A phase 2 study evaluating the VMAT simultaneous integrated boost (SIB) technique with a total dose of 57.5 Gy showed excellent pathological response, though high-grade toxicity was observed in over one-third of patients [11]. Despite this, all patients had negative resection margins, with one-year, three-year, and five-year disease-free survival (DFS) rates of 88.9%, 66.7%, and 66.7%, respectively [11].

Only sporadic studies have compared standard 3D-CRT with novel RT techniques in LARC treatment. A retrospective study comparing VMAT–SIB with 3D conformal therapy showed marginally improved pathological response rates (44% vs. 60%, *p* = 0.77) [12]. Limitations of current research include small patient groups, often single-center designs, and heterogeneity in radiation doses and chemotherapy regimens. Furthermore, most published studies included simultaneous modifications to RT doses and chemotherapy regimens, making it difficult to isolate the therapeutic effects and safety of dose-escalated RT compared to the standard approach [8].

The primary objective of this study was to compare the pCR rate between patients treated with the VMAT–SIB and 3D-CRT techniques in nCRT for rectal cancer. Secondary objectives included comparisons of acute toxicities, surgical characteristics (sphincter preservation rates and resection margin status), and tumor histopathological features after surgery (lymphovascular, vascular, and perineural invasion). Additionally, treatment efficacy was assessed by analyzing pathological stages, regression grades, downsizing, and downstaging.

## 2. Materials and Methods

This study was conducted at the Institute for Oncology and Radiology of Serbia (IORS) as a cohort study employing a mixed design. The dose-escalation group treatment with VMAT–SIB was conducted prospectively from June 2020 to January 2022, including 75 patients with LARC who received nCRT with a SIB technique. The comparison group was retrospective, consisting of 62 patients treated with a standard nCRT regimen using the 3D-CRT technique between 2018 and 2019. Baseline characteristics of patients are presented in Table 1.

The study was approved by the Ethics Committee of the IORS (Approval No. 2211-01 from 11 June 2020) and Ethics Committee of the Faculty of Medicine, University of Belgrade (Approval No. 1322/XII-17 from 3 December 2020). All patients signed their informed consent. The study was conducted in full compliance with the principles outlined in the Declaration of Helsinki, adhering to local legislation and institutional requirements to ensure that the highest ethical standards were maintained throughout the research process.

### 2.1. Patient Selection

**Figure 1:**
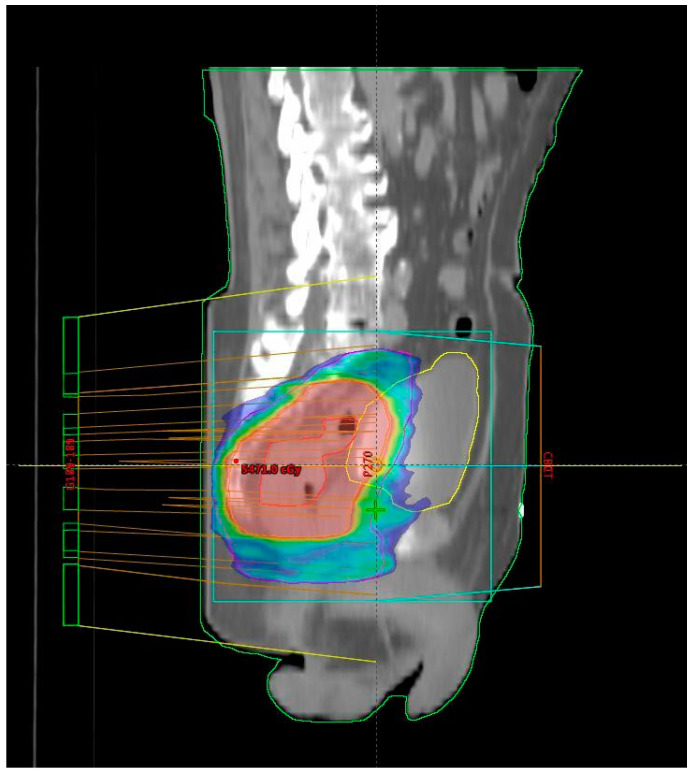
Isodose distribution of the volumetric modulated arc therapy (VMAT) radiotherapy plan—sagittal plane of computed tomography used for treatment planning (CT simulation). The central red zone corresponds to the simultaneous integrated boost (SIB) volume with a 51.75 Gy isodose, the green-blue area represents the planning target volume (PTV) coverage with a 42.75 Gy isodose, the yellow line outlines the bladder volume, and the red line delineates the gross tumor volume (GTV).

Patients included in the study were those with LARC who underwent nCRT using the VMAT–SIB technique, representing the prospective group treated with a novel RT technique. Figure 1 shows the isodose distribution of the VMAT plan. The total RT dose delivered was 54 Gy in 25 fractions, accompanied by concurrent CT following the 5-fluorouracil–leucovorin (5-FU–LV) protocol during the first and fifth weeks of RT. Inclusion criteria for the study were age over 18 years, clinically and histopathologically confirmed diagnosis of rectal adenocarcinoma, locally advanced disease stage (Stage II (T3/4N0M0) and III (T1-4N + M0) according to the UICC TNM classification, 8th edition) [13], tumor distal pole located within 12 cm from the anal verge, patients undergoing initial specific oncological treatment for rectal cancer (excluding colostomy, which was performed only in cases of intraluminal bowel obstruction), ECOG performance status of 0, 1, or 2 [14], adequate bone marrow function (leukocytes > 3.5 × 10^9^/L, neutrophils > 1.5 × 10^9^/L, platelets > 100 × 10^9^/L, hemoglobin > 80 g/L), satisfactory liver function (AST, ALT, bilirubin ≤ three times the upper limit of normal), creatinine clearance > 30 mL/min, understanding of the study protocol, and signed informed consent for participation. There were no restrictions based on age or comorbidities beyond those specifically related to liver and kidney function, as stated above. Exclusion criteria included a diagnosis of another malignancy within the last five years, previous treatment for rectal cancer, prior pelvic RT, allergy to fluoropyrimidines, active inflammatory bowel disease, uncontrolled hypertension, pregnancy or breastfeeding, failure to sign the informed consent, and not meeting the inclusion criteria.

To compare treatment outcomes and toxicity between the approach involving an escalated RT dose with SIB and the standard nCRT, a group of patients treated with standard therapy was also observed. Patients for the retrospective cohort were selected from the IORS institutional database based on identical inclusion and exclusion criteria to those used for the group treated with the VMAT–SIB approach. By applying consistent criteria across both cohorts, potential selection biases were mitigated. The standard RT approach involved treatment planning with 3D-CRT. The total RT dose was 50.4 Gy in 28 fractions, with concurrent CT following the 5-FU–LV protocol during the first and fifth weeks of RT. The CT protocol was identical for both groups, with the difference between the two groups being the RT planning technique and the total delivered RT dose.

### 2.2. Neoadjuvant Chemoradiotherapy

**Figure 2:**
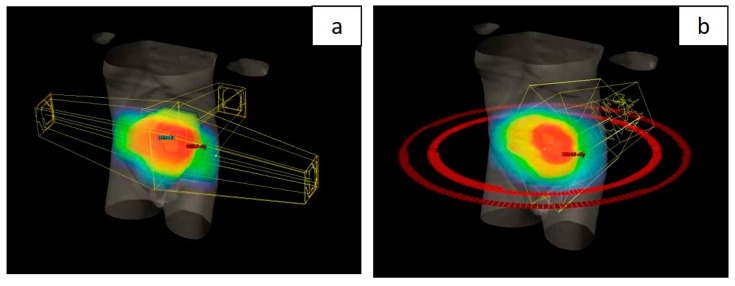
Comparative 3D visualization of radiotherapy planning techniques for locally advanced rectal cancer: 3D conformal technique (**a**) and volumetric modulated arc therapy (**b**).

All patients were treated using a long-course RT regimen. Radiotherapy planning for the group treated with the novel RT technique was performed using VMAT–SIB (Figure 2). Radiotherapy was delivered in 25 fractions over a period of five weeks. A tumor dose of 45 Gy (1.8 Gy per fraction) was applied to the mesorectal area and regional lymph nodes. An SIB was delivered to the area of macroscopic disease visible on imaging, with a 2 cm margin in all directions, at a total dose of 54 Gy (2.16 Gy per fraction). Concurrent CT included 5-FU at a dose of 350 mg/m^2^ and LV at a dose of 25 mg/m^2^. Chemotherapy was administered during the first and fifth weeks of RT.

Delineation of target volumes followed international protocols [15]. Tumor volumes were defined based on computed tomography simulation findings, pelvic magnetic resonance imaging (MRI) scans, and colonoscopy results. The gross tumor volume (GTV) and nodal GTV (GTVn) were outlined by visualizing the disease on each computed tomography slice. The GTV encompassed the full rectal wall thickness at the tumor site. The clinical target volume (CTV) included the GTV with a 2 cm margin in all directions, adjusted to fully encompass the mesorectum. The anterior CTV boundary accounted for physiological variations in pelvic organ positioning. Depending on tumor stage and localization, the CTV was further extended to the presacral space, lateral, external iliac, ischiorectal fossa, sphincter complex, and inguinal lymph nodes, divided into compartments for precision. Regardless of these factors, the mesorectum, posterior lateral nodes, and pelvic presacral nodes were always included in the CTV. The planning target volume (PTV) was defined by adding a 1 cm uniform margin around the CTV. For inguinal lymph nodes, adjustments were made to ensure a 3 mm distance from the skin. For simultaneous integrated boost (PTV boost), an additional 2 cm uniform margin around the GTV was applied, including pathologic nodes. Organs at risk (OARs) included the bladder (V50 ≤ 45%), small intestine (V45 ˂ 195 cm^3^), sigmoid colon (V50 < 50%), and femoral heads (V40 ˂ 40%; V45 ˂ 25%). The dose prescribed for the PTV was 45 Gy in 25 fractions, and for the PTV boost, 54 Gy in 25 fractions. Coverage criteria mandated that at least 95% of the PTV and PTV boost volumes receive 95% of the prescribed dose, with no more than 5% of the volume receiving 105% of the prescribed dose. Before initiation of the radiation treatment, dosimetric verification of the VMAT plan was performed. The radiation therapy was delivered using linear accelerators with 6X photon energy, in accordance with International Commission on Radiation Units and Measurements (ICRU) 62/83 protocols [16,17]. During the course of treatment, radiation precision was verified by comparing the current position obtained through megavoltage/kilovoltage/cone beam computed tomography (CBCT) imaging (portal imaging) with the digitally reconstructed radiograph (DRR) or computed tomography simulation as the reference position. This verification was conducted during the first radiation fraction and then at least once a week. A permissible displacement of up to 5 mm from the reference geometry was defined.

In contrast to the group treated with the VMAT–SIB technique, in which a higher RT dose was applied to the macroscopic disease volume, the group of patients treated with standard nCRT underwent a sequential boost technique. In this approach, after delivery of a TD of 45 Gy in 25 fractions (1.8 Gy per fraction), an additional RT dose of 5.4 Gy in three fractions (1.8 Gy per fraction) was applied to the volume of macroscopic disease. The CT protocol was the same as that used for the group treated with the VMAT–SIB technique. The target volumes defined were GTV, CTV, PTV, and PTV boost, according to the same protocols as in the group treated with the VMAT–SIB technique. Organs at risk (OARs) included the volumes of the bladder, small intestine, sigmoid colon, and femoral head. A dose of 45 Gy in 25 fractions (1.8 Gy per fraction) was prescribed for the PTV, and a dose with a TD of 5.4 Gy in 3 fractions (1.8 Gy per fraction) was prescribed for the PTV boost. Coverage criteria for the target volume were defined such that at least 95% of the PTV and PTV boost volumes should receive 95% of the prescribed dose, with no more than 5% of the PTV and PTV boost volumes receiving 105% of the prescribed dose. Dose limits for OARs did not differ from those for the cohort treated with the new radiation therapy technique. Planning was performed using the 3D-CRT technique (Figure 2) with three or four radiation fields. Radiation therapy was delivered using linear accelerators with photon energy of 15 and 18 MeV, in accordance with ICRU 50/62 protocols [16,18]. During the treatment, radiation precision was verified by comparing the current position using megavoltage imaging (portal imaging) with the DRR during the first radiation fraction and then weekly. A permissible displacement of up to 5 mm from the reference geometry was defined.

The full planned radiation dose and two cycles of chemotherapy were administered to all patients in both groups.

### 2.3. Acute Treatment-Related Complications

During the course of neoadjuvant treatment, the occurrence of acute complications was recorded. Acute gastrointestinal, urinary, dermatological, and hematological toxicities were monitored. The grading of complications was performed according to version 5.0 of the NCI CTCAE—NCI Common Terminology Criteria for Adverse Events [19].

### 2.4. Surgery

Surgical treatment, depending on the location of the disease, was performed using either abdominoperineal resection according to Miles or low anterior resection of the rectum with total mesorectal excision (resectio recti anterior inferior—RRAI). After the completion of nCRT in the prospective group, among 63 patients who underwent surgery, 37 (59%) had their surgery at the Clinic for Digestive Surgery, University Clinical Center, Belgrade, while 26 patients (41%) underwent surgery at the IORS, Belgrade. In the retrospective group, among 56 patients who had surgery, 44 (78.6%) had their surgery at the Clinic for Digestive Surgery, and 12 patients (21.4%) underwent surgery at the IORS. For patients with documented clinical complete response (cCR), for whom, due to the distal location of the disease, sphincter-preserving surgery was not possible, a non-operative approach (“watch and wait” approach) was suggested.

### 2.5. Analyzed Treatment Outcomes

Data collection included comprehensive demographic and clinical characteristics of patients, such as age, gender, tumor localization, disease staging, and baseline tumor marker levels (CEA and CA 19-9). Detailed treatment parameters were recorded, including radiation doses, chemotherapy regimens, and surgical procedures performed.

The analysis of surgical treatment characteristics included a comparison of the two study groups in terms of the percentage of patients who underwent surgery after completing nCRT, the timing of surgery following the conclusion of neoadjuvant treatment, the percentage of sphincter-preserving surgeries, and the status of resection margins. The groups were also compared regarding the histopathological characteristics of the tumor after surgery, including the grade of differentiation, presence and percentage of mucinous tumor component, and the presence of lymphovascular invasion (LVI), vascular invasion (VI), and perineural invasion (PNI).

Definitive pathological staging of the disease (T category, N category, and disease stage) was also assessed for both treatment approaches. Patients with a good response to neoadjuvant treatment were defined as those with T0, T1, or T2 in the postoperative T category and those with localized disease (pCR, 1, or 2) in the postoperative stage. Additionally, differences in the N category, including the total number of lymph nodes examined and the number of positive lymph nodes, were analyzed. The groups were compared using Dukes and Astler–Coller disease staging systems. In both groups, pathological response to treatment was assessed using the tumor regression grade (TRG) classification by Mandard and the rectal cancer regression grade (RCRG) classification. Differences between the two groups in relation to these outcomes were analyzed. Some patients with cCR and initially distantly located tumors were managed non-surgically and enrolled in a strict follow-up program as part of the “watch and wait” approach.

Reduction in T and N categories, as well as disease stage, was calculated based on initial parameters before starting neoadjuvant treatment and the definitive pathological stage of the disease. As measures of treatment efficacy, the two groups were compared in terms of reduction in the T category, reduction in the N category, reduction in either the T or N category, and reduction in disease stage.

### 2.6. Comparability of the Two Study Groups

As an initial step, a comparison was made between the group of patients treated with the new RT technique and the group treated with the standard approach. This comparison focused on patient characteristics (sex, age, ECOG performance status, body mass index (BMI)), tumor characteristics (localization, length of the affected intestinal segment, differentiation grade, T and N category, disease stage), and baseline tumor marker values (CEA, CA 19-9). By eliminating significant differences, conditions were established for a valid comparison and accurate interpretation of differences in recorded acute toxicity and treatment outcomes between the two study groups.

### 2.7. Study Endpoints

The study compared the group of patients treated with the VMAT–SIB (54 Gy in 25 fractions) and 3D-CRT technique (50.4 Gy in 28 fractions) according to primary and secondary endpoints. The primary endpoint was the pCR rate. Secondary endpoints included acute toxicity rates, surgical outcomes, and histopathological tumor characteristics. Surgical outcomes analyzed were sphincter-preservation rates and resection margin status. Histopathological analysis included LVI, VI, and PNI statuses. Treatment efficacy measures included tumor pathological stages, regression grades (Mandard TRG, RCRG), T and N category reduction, and disease downstaging.

### 2.8. Statistical Analyses

For testing normal data distribution, the Kolmogorov–Smirnov and Shapiro–Wilk tests were used. Descriptive statistical methods (frequencies, percentages, mean, median, standard deviation (SD), and range) were applied to summarize the data. The statistical significance level (alpha value) was set at *p* < 0.05. To compare disease characteristics, treatment details, toxicities, and outcomes between the two patient groups, statistical tests were selected based on data type and distribution: the Wilcoxon rank-sum test for continuous variables, and Pearson’s chi-square test or Fisher’s exact test for categorical variables. To control for potential confounding factors, the two patient groups were compared based on demographics and clinical characteristics before conducting statistical tests to evaluate outcomes. All statistical analyses were conducted using the R programming environment (version 3.3.2, released on October 31, 2016—“Sincere Pumpkin Patch”; Copyright © 2016 The R Foundation for Statistical Computing; Platform: x86_64-w64-mingw32/x64 (64-bit); downloaded on 21 June 2024).

## 3. Results

Comparing baseline characteristics of the two patient groups revealed no statistically significant differences in sex, age, or BMI (Table 1). Both groups comprised patients in good general condition before treatment initiation (ECOG PS 0 or 1). A higher proportion of patients with ECOG PS 0 status was observed in the standard nCRT group. Regarding tumor characteristics, a significantly higher number of patients in the VMAT–SIB group had distally located rectal cancer (*p* < 0.01). No statistically significant differences were found in the initial tumor marker levels between the groups.

The comparison of acute toxicity observed in the two groups is presented in Table 2 and Supplementary Table S1. Urinary toxicity was significantly more frequent in the group of patients treated with the new RT technique compared to the group treated with the standard approach (*p* < 0.01). Other complications were similarly distributed between the two treatment approaches. Regarding the timing of acute toxicity onset, it occurred earlier in the group of patients treated with the new RT technique (*p* < 0.01).

In the group of patients treated with the novel RT technique, the average time to surgery after nCRT was 14.1 weeks, compared to 12.3 weeks in the group treated with the standard approach. This difference was statistically significant (*p* < 0.01, Table 3, Supplementary Table S2). No statistically significant differences were observed between the groups regarding other surgical characteristics.

Comparison of tumor characteristics after surgical treatment revealed a significantly higher presence of perineural invasion in the group of patients treated with the standard approach (Table 4, Supplementary Table S3).

In terms of the final pathohistological stage of disease, a significantly higher prevalence of lower T categories (T0, T1, T2; *p* < 0.01), N-negative categories (*p* < 0.05), and lower disease stages (I, II; *p* < 0.05) was observed in the group of patients treated with the new RT technique (Table 5, Supplementary Table S4). A comparison of the number of examined lymph nodes revealed a statistically significant higher number of examined lymph nodes in the group of patients treated with the standard approach (*p* < 0.05).

A statistically significant difference in response was observed between the two treatment methods (Table 6, Supplementary Table S5). A significantly higher representation of lower TRG and RCRG scores was found in the group of patients treated with the new radiation therapy technique. However, when comparing pathological complete response (TRG1) with all other categories, a positive trend was noted (*p* = 0.06), but without statistical significance.

A statistically significantly higher percentage of reduction in the T category of disease, as well as disease stage (*p* < 0.01), was observed in the group of patients treated with the new radiation therapy technique (Table 7, Supplementary Table S6).

## 4. Discussion

Optimal chemoradiotherapy approaches, the appropriate timing for response evaluation of LARC after nCRT, the ideal timing for surgery, and the criteria for selecting the best candidates for delaying surgery with intensive follow-up (“watch and wait” approach) remain unclear. In our previous studies, we explored potential predictors of response to nCRT based on clinical, radiological, and molecular observations [20,21,22]. The aim of our study was to compare a group of patients treated with a new radiation therapy technique and a group treated with standard CRT as part of neoadjuvant treatment for LARC, with the goal of evaluating the benefits of dose escalation using an SIB technique. In the standard treatment group, RT involved a sequential boost to the area of macroscopic disease, achieving a total dose of 50.4 Gy in 28 fractions using the 3D-CRT technique. In contrast, the new technique applied VMAT with SIB to the area of macroscopic disease, delivering a total dose of 54 Gy in 25 fractions. It is important to note that both groups received the same CT regimen. Before analyzing differences in toxicity and treatment outcomes, a comparison of the two groups was conducted based on their demographic and disease characteristics. As no significant differences were observed, comparability between the groups was ensured.

Our results showed a significantly higher number of non-hematological toxicities in the group of patients treated with the new radiation therapy technique. However, when toxicities were classified as low-grade (Grade 1 and 2) or high-grade (Grade 3), no significant differences were observed between the groups. Despite the use of the VMAT technique, known for its superior conformality and ability to spare surrounding OARs, toxicity rates were similar between the two groups of patients. This unexpected outcome may be attributed to several factors. First, the higher dose per fraction (2.16 Gy) and the greater total RT dose (54 Gy vs. 50.4 Gy) in the group of patients treated with the new technique could have offset the benefits of the more conformal approach regarding toxicity rates. Additionally, the prospective nature of the group treated with the new technique and stricter assessment of low-grade toxicities may have revealed adverse effects that were not documented in the group treated with the standard approach.

In a study conducted by Bae et al., comparing the use of a boost dose delivered sequentially with the 3D conformal technique and simultaneously with the IMRT technique, a significantly lower rate of urinary toxicity was observed in the IMRT–SIB group (patients with no toxicity or Grade 1 toxicity were compared to those with Grade 3 toxicity). However, no significant differences were found in other parameters [23]. The difference compared to our study lies in the absence of an increased RT dose in the IMRT–SIB group, enabling a comparison of techniques without the confounding effect of the total RT dose.

Previous studies aiming to determine gastrointestinal toxicity rates also did not report reduced toxicity with the use of newer RT planning techniques. Gastrointestinal toxicity was observed in 52.5% of patients, which aligns with the findings of our study [24]. In a study comparing conventional and VMAT–SIB techniques with an RT dose of 45 Gy and a boost to a total dose of 55 Gy, Grade 2 urinary toxicity was reported in 49% of patients, while Grade 2 diarrhea was reported in 42% of patients [12]. Similar toxicity results were observed in a study conducted by Yang et al., which utilized the VMAT–SIB technique (50 Gy/58.75 Gy in 25 fractions). Most patients experienced some form of acute toxicity, with a high-grade toxicity rate (Grade 3) of 7.7% [8].

Further investigation into specific types of toxicities in our study revealed significant differences in urinary system toxicities, with a higher incidence of dysuria and frequent urination observed in the group of patients treated with the new RT technique. The predominant localization of tumors in the distal rectum in this group was associated with higher doses delivered to the urethra due to its close proximity to the mesorectum in this region.

Analysis of toxicity onset during treatment showed significantly earlier occurrence in the group of patients treated with the new RT technique. This difference may be attributed to the higher dose per fraction (2.16 Gy) delivered to the macroscopic tumor area. Notably, this analysis was not part of the data reported in the literature, making our findings a valuable contribution to understanding the timing of toxicity onset. These insights allow for the timely implementation of supportive measures to minimize adverse reactions.

Among the operated patients, sphincter-preserving surgery was achieved in 49 out of 63 in the group treated with the new RT technique, representing a higher percentage (77.8%) compared to 35 out of 56 patients in the standard approach (62.5%). However, statistical significance was not achieved. The lack of statistical significance could be attributed to the higher proportion of distally located tumors in the group treated with the new RT technique. Importantly, in 12 patients with distal tumor localization, achieving a cCR allowed for a non-operative management approach, resulting in sphincter preservation. Our findings align with the existing literature, in which a study utilizing the VMAT–SIB technique (45 Gy/55 Gy) reported a sphincter preservation rate of 88%. However, that study had a higher initial prevalence of stage II disease (42%) compared to our study [12].

Comparison of the two patient groups revealed a statistically significant lower incidence of PNI in postoperative specimens from patients treated with the new RT technique (*p* < 0.01). This finding is important given the well-established negative prognostic impact of PNI in colorectal cancer. A meta-analysis of 58 studies involving 22,900 colorectal cancer patients demonstrated that PNI is associated with worse five-year overall survival (OS) (relative risk (RR), 2.09; 95% confidence interval, 1.68–2.61) and DFS (RR, 2.35; 95% CI, 1.66–3.31) [25].

Analysis of definitive postoperative staging showed significantly higher rates of lower T categories (T0–2), negative nodal status (N0), and earlier stages (I + II) in the group treated with the new RT technique. Literature comparisons suggest that a change in RT technique alone, without dose escalation, does not result in significant improvements in these parameters [23]. The observed improvements in our study likely reflect the combined effect of VMAT–SIB and increased RT dose.

Literature data indicate that pCR is associated with improved outcomes, regardless of initial clinical T and N categories, highlighting its importance in assessing LARC response to nCRT [26]. In our study, among operated patients, pCR was achieved in 13 out of 63 patients (20.6%) treated with the new RT technique, compared to 5 out of 56 patients (8.9%) treated with the standard approach, showing a positive trend without statistical significance (*p* = 0.06). The lack of significance may be attributed to the 16% of patients in the new technique group who, due to cCR, did not undergo surgery. A study of 385 LARC patients treated with nCRT (50.4 Gy) followed by surgery after six weeks reported a pCR rate of 10.4% [27]. Similarly, a study employing the VMAT–SIB technique with dose escalation, like ours, observed a pCR rate of 17% [12], demonstrating the comparability of our results with the literature.

Tumor regression grade (TRG) is an important prognostic factor for assessing the risk of local recurrence, DFS, and OS in patients with LARC [28]. When comparing individual TRG statuses, significantly better treatment responses were noted in the group treated with the new RT technique. For RCRG classification, statistical significance was observed both for individual categories and when comparing patients with favorable (RCRG1) vs. poor responses (RCRG2–3). The divergence between classifications arises from the lack of standardized response alignment in the literature. Extended follow-up will help determine which classification has greater prognostic value in our patient cohort. Research indicates that ypN status and TRG status are independent predictors of OS and recurrence-free survival (RFS). A large study of 237 LARC patients treated with nCRT highlighted the importance of these predictors [29]. In our comparison of two groups, a statistically significant lower percentage of N-negative cases was observed in the group treated with the new RT technique. Understanding the prognostic value of N status after neoadjuvant treatment can support an individualized approach to intensifying adjuvant chemotherapy in high-risk patient groups.

Guidelines recommend examining at least 12 lymph nodes for accurate staging of rectal cancer [30]. However, when treatment begins with nCRT, achieving this benchmark can be challenging, as neoadjuvant therapy often reduces both the size and number of lymph nodes in the treated area. Our findings align with the literature, in which intensified neoadjuvant treatment in the group treated with the new RT technique has been associated with a statistically significant reduction in the number of isolated lymph nodes. A study comparing outcomes based on lymph node evaluation (fewer than 12 vs. 12 or more examined) found a higher percentage of pCR in patients with fewer than 12 isolated lymph nodes [31]. The exact number of lymph nodes needed for staging in nCRT-treated patients remains undefined. Additional studies show that the number of isolated lymph nodes can vary with factors such as gender, age, initial disease stage, and the presence of LVI. Interestingly, a smaller number of isolated lymph nodes is often associated with better tumor response and improved prognosis [32].

Data from the literature indicate that response to neoadjuvant therapy strongly correlates with long-term treatment outcomes. Following neoadjuvant treatment, disease downstaging is achieved in 50–60% of patients, while approximately 20% achieve pCR [33,34]. Unlike most studies that evaluate the pathological response to neoadjuvant treatment independently of ypTN staging, some approaches emphasize incorporating disease stage into the assessment. It has been demonstrated that the prognostic value of response is particularly significant in patients with ypIII disease. In this group, those with a good response to nCRT and a ypIII stage show survival rates comparable to those with ypII disease (five-year OS: 67% vs. 74%, *p* = 0.89). Conversely, in patients with a poor pathological response within the ypIII stage, survival outcomes resemble those of patients with stage IV disease (five-year OS: 27% vs. 18%, *p* = 0.09) [35].

This study has certain limitations. The sample size is relatively small but meets the minimum criteria for patients with LARC, considering its prevalence and the population size in Serbia (95% confidence level) [36]. In comparing the approach involving dose escalation with SIB using the VMAT technique against the standard treatment, limitations include the retrospective nature of the comparator group, leading to incomplete data on toxicity and treatment outcomes. Additionally, the study’s single-institution design may limit the generalizability of the findings. However, our institution, the IORS, is the national cancer research and treatment center and, as such, receives a high load of patients from all over the country, and is thus representative of the national situation. Other limitations may include potential selection bias due to the retrospective analysis and the lack of long-term follow-up data, which would provide a more comprehensive understanding of survival and recurrence outcomes, as well as late toxicity rates. One of the limitations of this study is the higher proportion of distally located tumors in the VMAT–SIB group (80%) compared to the 3D-CRT group (50%), which could potentially influence the sphincter preservation rates. This difference in baseline tumor location makes direct comparisons between the groups more challenging. While sphincter-preserving surgery was achieved in a higher percentage of patients in the VMAT–SIB group, the lack of statistical significance may be attributed to this baseline disparity in tumor location. Thus, the results should be interpreted with caution, and further studies are needed to better understand the impact of tumor location on the outcomes of dose escalation in radiotherapy.

To address these limitations, future multi-institutional, prospective studies with larger sample sizes are planned to confirm these findings and further validate the results. Conducting a randomized controlled trial (RCT) is identified as a crucial step to provide stronger evidence. This approach has been proposed within the framework of the STEPUPIORS Horizon Europe project (“https://cordis.europa.eu/project/id/101079217 (accessed on 10 February 2025)”), which involves collaboration among four European countries. The mixed design (prospective–retrospective) used in this study served as the best available approach given the data and patient population in Serbia, offering a real-world data pilot for a future RCT. Multi-center validation is considered essential to provide a broader perspective and enhance the clinical applicability of the results. This validation is planned within the STEPUPIORS consortium, involving four research institutions dedicated to rectal cancer and other European partners established during the project’s implementation. Further multi-institutional studies are expected to provide statistical confirmation of these findings and strengthen the conclusions. These efforts will follow this study, which primarily focused on Serbian patients. Additionally, as these findings are the first of their kind for the Slavic population, comparisons with other populations within the consortium may reveal population-specific effects, contributing valuable insights.

## 5. Conclusions

This study demonstrates the potential benefits of dose-escalated RT using the VMAT–SIB technique in the treatment of patients with LARC. Improved treatment outcomes, including higher rates of pCR and favorable TRG responses, were observed in the group of patients treated with the novel technique. Overall, this study supports the integration of dose-escalated VMAT–SIB into clinical practice as a feasible and effective approach for managing LARC, contributing to a more personalized and effective therapeutic strategy. However, further prospective, multi-center studies are needed to validate these results and assess long-term outcomes.

## Figures and Tables

**Table 1:** Baseline characteristics of patients and disease in the two groups of patients.

Characteristics	Study Group		
	VMAT–SIB ^c^	3D-CRT ^d^	Wilcoxon Rank-Sum Test
**Gender**			
Male	50 (66.7%)	43 (69.4%)	ns ^f^
Female	25 (33.3%)	19 (30.6%)	
**Age (years)**			
Mean (SD)	60.8 (10.6)	59.4 (10.2)	ns
Median (Range)	62 (33–81)	60 (29–76)	
**Performance status (PS) ^a^**			
ECOG PS 0	52 (69.3%)	58 (93.5%)	*p* < 0.01 ^g^
ECOG PS 1	23 (30.7%)	4 (6.6%)	
**Body mass index**			
Mean (SD)	26.7 (4.7)	25.8 (4.1)	ns
Median (Range)	26.4 (18.6–41.0)	25.5 (16.7–41.6)	
**Tumor location ^e^ (cm)**			
Lower (anal verge ≤ 8)	60 (80.0%)	31 (50.0%)	*p* < 0.01 ^f^
Middle (8 < anal verge ≤ 12)	15 (20.0%)	31 (50.0%)	
**Tumor length (mm)**			
Mean (SD)	63.2 (18.6)	57.8 (15.1)	ns
Median (Range)	60 (24–150)	56 (26–100)	
**Tumor differentiation**			
Well and moderate	69 (92.0%)	59 (95.2%)	ns ^g^
Poor	6 (8.0%)	3 (4.8%)	
**T in clinical TNM**			
T2	2 (2.7%)	1 (1.6%)	ns ^g^
T3	64 (85.3%)	56 (90.3%)	
T4	9 (12.0%)	5 (8.1%)	
**N in clinical TNM**			
N0	1 (1.3%)	3 (4.8%)	ns ^g^
N1	22 (29.3%)	18 (29.0%)	
N2	52 (69.4%)	41 (66.1%)	
**UICC ^b^ staging**			
II	1 (1.3%)	3 (4.8%)	ns ^g^
III	74 (98.7%)	59 (95.2%)	
**CEA (µg/mL)**			
Mean (SD)	9.8 (22.3)	6.2 (10.4)	ns
Median (Range)	3.5 (0.6–167.0)	2.9 (0.1–58.0)	
**CA 19-9 (µg/mL)**			
Mean (SD)	14.6 (23.1)	14.6 (18.4)	ns
Median (Range)	8.0 (0.8–118.0)	9.1 (0.0–107.0)	
**Total**	**75 (100%)**	**62 (100%)**	***-***

^a^ ECOG PS, Eastern Cooperative Oncology Group performance status; ^b^ UICC, Union for International Cancer Control; ^c^ VMAT–SIB—group of patients treated with the new radiotherapy technique; ^d^ 3D-CRT—group of patients treated with the standard approach; ^e^ tumor location—distance from anal verge; ^f^ Pearson χ^2^ test; ^g^ Fisher exact test; ns—not statistically significant.

**Table 2:** Acute toxicity profile in the two groups of patients.

Characteristics	Study Group		
	VMAT–SIB ^a^	3D-CRT ^b^	Wilcoxon Rank-Sum Test
**Acute toxicity**			
No	4 (5.3%)	9 (14.5%)	ns ^d^
Yes	71 (94.7%)	53 (85.5%)	
**Acute haematological toxicity**			
No	51 (68.0%)	47 (75.8%)	ns ^c^
Yes	24 (32.0%)	15 (24.2%)	
**Acute non-haematological toxicity**			
No	4 (5.3%)	12 (19.4%)	*p* < 0.01 ^d^
Yes	71 (94.7%)	50 (80.6%)	
**Radiodermatitis**			
No	22 (29.3%)	16 (25.8%)	ns ^d^
Grade 1 and 2	49 (65.3%)	43 (69.3%)	
Grade 3	4 (5.3%)	3 (4.8%)	
**Diarrhea**			
No	43 (57.3%)	39 (62.9%)	ns ^d^
Grade 1 and 2	29 (38.7%)	23 (37.1%)	
Grade 3	3 (4%)	0 (0%)	
**Urinary toxicity—dysuria**			
No	44 (58.7%)	49 (79.0%)	*p* < 0.05 ^c^
Yes	31 (41.3%)	13 (21.0%)	
**Urinary toxicity—frequent urination**			
No	48 (64.0%)	55 (88.7%)	*p* < 0.01 ^c^
Yes	27 (36.0%)	7 (11.3%)	
**Nausea**			
No	60 (80.0%)	53 (85.5%)	ns ^c^
Grade 1 and 2	15 (20.0%)	9 (14.5%)	
**Vomiting**			
No	71 (94.7%)	59 (95.2%)	ns ^d^
Grade 1 and 2	4 (5.3%)	3 (4.8%)	
**Week of reported acute toxicity**			
Mean (SD)	3.1 (1.0)	3.6 (0.7)	*p* < 0.01
Median (Range)	3 (2–6)	4 (2–5)	
**Total**	**75 (100%)**	**62 (100%)**	-

^a^ VMAT–SIB—group of patients treated with the new radiotherapy technique; ^b^ 3D-CRT—group of patients treated with the standard approach; ^c^ Pearson χ^2^ test; ^d^ Fisher exact test; ns—not statistically significant.

**Table 3:** Surgical characteristics in the two patient groups.

Characteristics	Study Group		
	VMAT–SIB ^a^	3D-CRT ^b^	Wilcoxon Rank-Sum Test
**Surgery**			
No	12 (16.0%)	6 (9.7%)	ns ^c^
Yes	63 (84.0%)	56 (90.3%)	
**Time to surgery after completing neoadjuvant treatment (weeks)**			
Mean (SD)	14.1 (4.5)	12.3 (6.7)	*p* < 0.01
Median (Range)	13 (9–35)	10 (5–39)	
**Type of surgery**			
Abdominoperineal resection	14 (18.7%)	21 (33.9%)	ns ^d^
Sphincter-sparing surgery	49 (65.3%)	35 (56.4%)	
Not operated	12 (16.0%)	6 (9.7%)	
**Resection margin**			
Negative	58 (77.3%)	50 (80.6%)	ns ^d^
Positive	5 (6.7%)	6 (9.7%)	
Not operated	12 (16.0%)	6 (9.7%)	
**Total**	**75 (100%)**	**62 (100%)**	**-**

^a^ VMAT–SIB—group of patients treated with the new radiotherapy technique; ^b^ 3D-CRT—group of patients treated with the standard approach; ^c^ Pearson χ^2^ test; ^d^ Fisher exact test; ns—not statistically significant.

**Table 4:** Comparison of pathohistological tumor characteristics between the two patient groups.

Characteristics	Study Group		
	VMAT–SIB ^a^	3D-CRT ^b^	Pearson χ^2^ Test
**Degree of tumor differentiation**			
Grade 1/2	42 (56.0%)	48 (77.5%)	ns ^c^
Grade 3	4 (5.3%)	3 (4.8%)	
NA/Not operated	29 (38.7%)	11 (17.7%)	
**Presence of mucinous tumor component**			
No	51 (68.0%)	41 (66.1%)	ns ^c^
Yes	12 (16.0%)	14 (22.6%)	
NA/Not operated	12 (16.0%)	7 (11.3%)	
**Lymphovascular invasion**			
No	48 (64.0%)	35 (56.4%)	ns
Yes	14 (18.7%)	21 (33.9%)	
NA/Not operated	13 (17.3%)	6 (9.7%)	
**Vascular invasion**			
No	54 (72.0%)	44 (71.0%)	ns
Yes	7 (9.3%)	12 (19.3%)	
NA/Not operated	14 (18.7%)	6 (9.7%)	
**Perineural invasion**			
No	57 (76.0%)	38 (61.3%)	*p* < 0.01
Yes	6 (8.0%)	17 (27.4%)	
NA/Not operated	12 (16.0%)	7 (11.3%)	
**Total**	**75 (100%)**	**62 (100%)**	***-***

^a^ VMAT–SIB—group of patients treated with the new radiotherapy technique; ^b^ 3D-CRT—group of patients treated with the standard approach; ^c^ Fisher exact test; ns—not statistically significant; NA—data not available.

**Table 5:** Comparison of pathohistological stage of disease between the two patient groups.

Characteristics	Study Group		
	VMAT–SIB ^a^	3D-CRT ^b^	Wilcoxon Rank-Sum Test
**T in pathological TNM**			
T0 + T1 + T2	41 (54.7%)	18 (29.0%)	*p* < 0.01 ^c^
T3 + T4	22 (29.3%)	38 (61.3%)	
Not operated/cCR ^e^	12 (16.0%)	6 (9.7%)	
**N in pathological TNM**			
N0	49 (65.3%)	32 (51.6%)	*p* < 0.05 ^c^
N1 + N2	14 (18.7%)	24 (38.7%)	
Not operated/cCR ^e^	12 (16.0%)	6 (9.7%)	
**Number of examined lymph nodes**			
Mean (SD)	13.1 (6.0)	16.9 (9.3)	*p* < 0.05
Median (Range)	13 (3–31)	16 (0–45)	
**Number of positive lymph nodes**			
Mean (SD)	0.6 (1.5)	1.3 (2.5)	*p* < 0.05
Median (Range)	0 (0–7)	0 (0–12)	
**UICC ^d^ staging**			
pCR ^f^ + I + II	49 (65.3%)	32 (51.6%)	*p* < 0.05 ^c^
III	14 (18.7%)	24 (38.7%)	
Not operated/cCR ^e^	12 (16.0%)	6 (9.7%)	
**Dukes classification of the disease**			
pCR ^f^ + A + B	50 (66.7%)	31 (49.9%)	*p* < 0.01
C + D	13 (17.3%)	25 (40.3%)	
Not operated/cCR ^e^ (not compared)	12 (16.0%)	6 (9.8%)	
**Astler–Coller classification of the disease**			
pCR ^f^ + A + B1	33 (44.0%)	15 (24.1%)	*p* < 0.01
B2 + C + D	30 (40.0%)	41 (66.1%)	
Not operated/cCR ^e^ (not compared)	12 (16.0%)	6 (9.8%)	
**Total**	**75 (100%)**	**62 (100%)**	***-***

^a^ VMAT–SIB—group of patients treated with the new radiotherapy technique; ^b^ 3D-CRT—group of patients treated with the standard approach; ^c^ Pearson χ^2^ test; ns—not statistically significant; ^d^ UICC, Union for International Cancer Control; ^e^ cCR—clinical complete response; ^f^ pCR—pathological complete response (no defined Dukes and Astler–Coller classification of the disease).

**Table 6:** Comparison of the two patient groups regarding pathological response to treatment.

Characteristics	Study Group		
	VMAT–SIB ^a^	3D-CRT ^b^	Pearson χ^2^ Test
**Tumor regression grade (TRG)**			
TRG1	13 (17.3%)	5 (8.1%)	*p* < 0.05 ^c^
TRG2	10 (13.3%)	9 (14.5%)	
TRG3	30 (40.0%)	18 (29.0%)	
TRG4	10 (13.3%)	22 (35.5%)	
TRG5	0 (0%)	2 (3.2%)	
Not operated	12 (16.0%)	6 (9.7%)	
**TRG—groups**			
TRG1 (pCR)	13 (17.3%)	5 (8.1%)	*p* = 0.06 ^c^
TRG2-5	50 (66.7%)	51 (82.2%)	
Not operated	12 (16.0%)	6 (9.7%)	
**TRG—groups**			
TRG1-2	23 (30.7%)	14 (22.6%)	ns
TRG3-5	40 (53.3%)	42 (67.7%)	
Not operated	12 (16.0%)	6 (9.7%)	
**Rectal cancer regression grade (RCRG)**			
RCRG1	28 (37.3%)	12 (19.3%)	*p* < 0.05
RCRG2	24 (32.0%)	23 (37.1%)	
RCRG3	11 (14.7%)	21 (33.9%)	
Not operated	12 (16.0%)	6 (9.7%)	
**RCRG—groups**			
RCRG1	28 (37.3%)	12 (19.4%)	*p* < 0.01
RCRG2-3	35 (46.7%)	44 (71.0%)	
Not operated	12 (16.0%)	6 (9.7%)	
**Total**	**75 (100%)**	**62 (100%)**	*-*

^a^ VMAT–SIB—group of patients treated with the new radiotherapy technique; ^b^ 3D-CRT—group of patients treated with the standard approach; ^c^ Fisher exact test; ns—not statistically significant.

**Table 7:** Comparison of the two groups of patients in relation to the reduction in disease category and stage.

Characteristics	Study Group		
	VMAT–SIB ^a^	3D-CRT ^b^	Pearson χ^2^ Test
**Reduction of initial T stage**			
Yes	45 (60.0%)	22 (35.5%)	*p* < 0.01
No	18 (24.0%)	34 (54.8%)	
Not operated	12 (16.0%)	6 (9.7%)	
**Reduction of initial N stage**			
Yes	55 (73.3%)	44 (71.0%)	ns
No	8 (10.7%)	12 (19.3%)	
Not operated	12 (16.0%)	6 (9.7%)	
**Reduction of initial T or N stage**			
Yes	58 (77.3%)	46 (74.2%)	ns
No	5 (6.7%)	10 (16.1%)	
Not operated	12 (16.0%)	6 (9.7%)	
**Reduction of initial T and N stages**			
Yes	42 (56.0%)	20 (32.3%)	*p* < 0.01
No	21 (28.0%)	36 (58.1%)	
Not operated	12 (16.0%)	6 (9.7%)	
**Downstaging**			
Yes	50 (66.7%)	31 (50.0%)	*p* < 0.01
No	13 (17.3%)	25 (40.3%)	
Not operated	12 (16.0%)	6 (9.7%)	
**Total**	**75 (100%)**	**62 (100%)**	***-***

^a^ VMAT–SIB—group of patients treated with the new radiotherapy technique; ^b^ 3D-CRT—group of patients treated with the standard approach; ns—not statistically significant.

## Data Availability

The data that support the findings of this study are available upon reasonable request from the corresponding author. The data are not publicly available due to ethics restrictions, as the information contained could compromise the privacy of patients.

## Supplementary Materials

The following supporting information can be downloaded at: https://www.mdpi.com/article/10.3390/medicina61030483/s1. Table S1. Acute toxicity profile in the two groups of patients. Table S2. Surgical characteristics in the two patient groups. Table S3. Comparison of pathohistological tumor characteristics between the two patient groups. Table S4. Comparison of pathohistological stage of disease between the two patient groups. Table S5. Comparison of the two patient groups regarding pathological response to treatment. Table S6. Comparison of the two groups of patients in relation to the reduction in disease category and stage.

## Abbreviations

The following abbreviations are used in this manuscript:

LARC	locally advanced rectal cancer
nCRT	neoadjuvant chemoradiotherapy
RT	radiotherapy
CT	chemotherapy
pCR	pathological complete regression
CR	complete response
3D CRT	3D conformal radiation therapy
IMRT	intensity-modulated radiotherapy
VMAT	volumetric modulated arc therapy
TD	tumor dose
DFS	disease-free survival
SIB	simultaneous integrated boost
IORS	Institute for Oncology and Radiology of Serbia
MRI	magnetic resonance imaging
GTV	gross tumor volume
GTVn	nodal GTV
CTV	clinical target volume
PTV	planning target volume
CBCT	cone beam computed tomography
DRR	digitally reconstructed radiograph
ICRU	International Commission on Radiation Units and Measurements
RRAI	resectio recti anterior inferior
5-FU–LV	5-fluorouracil–leucovorin
LVI	lymphovascular invasion
VI	vascular invasion
PNI	perineural invasion
TRG	tumor regression grade
RCRG	rectal cancer regression grade
cCR	clinical complete response
SD	standard deviation
BMI	body mass index
OAR	organs at risk
OS	overall survival
RR	relative risk
RFS	recurrence-free survival
RCT	randomized controlled trial
