# Supplementary material for: Dose Escalation in Neoadjuvant Chemoradiotherapy for Rectal Cancer: Short-Term Efficacy and Toxicity of VMAT–SIB vs. 3D-CRT
Source: Medicina (Kaunas). 2025 Mar 11;61(3):483. doi: 10.3390/medicina61030483 (PMC11943629; doi:10.3390/medicina61030483)
Supplement: Supplementary file 1 [file medicina-61-00483-s001.zip › medicina-3483644-supplementary.pdf]

Supplementary Table S1. Acute toxicity profile in the two groups of patients.

Characteristics	Study group		Ratio (95%CI[d])
	VMAT-SIB[a]	3D CRT[b]	(VMAT-SIB[a])/(3D CRT[b])
Acute toxicity			
Yes	71/75	53/62	1.11 (0.99-1.24)
Acute haematological toxicity			
Yes	24/75	15/62	1.32 (0.76-2.29)
Acute non-haematological toxicity			
Yes	71/75	50/62	1.17 (1.03-1.34)
Radiodermatitis			
Yes	53/75	46/62	0.95 (0.77-1.17)
Diarrhoea			
Yes	32/75	23/62	1.15 (0.76-1.75)
Urinary toxicity - dysuria			
Yes	31/75	13/62	1.97 (1.13-3.43)
Urinary toxicity – frequent urination			
Yes	27/75	7/62	3.19 (1.49-6.82)
Nausea			
Yes	15/75	9/62	1.38 (0.65-2.93)
Vomiting			
Yes	4/75	3/62	1.1 (0.26-4.74)
Week of reported acute toxicity			
Mean (SD)	3.1 (1.0)	3.6 (0.7)	-0.5 (-1.42 - 0.42)[c]
Total	**75 (100%)**	**62 (100%)**	-

[a]VMAT-SIB - group of patients treated with the new radiotherapy technique; [b]3D CRT - group of patients treated with the standard approach; [c]difference between two mean values for VMAT-SIB[a] and 3D CRT[b] group; [d]CI – confidence interval

Supplementary Table S2. Surgical characteristics in the two patient groups.

Characteristics	Study group		Ratio (95%CI[c])
	VMAT-SIB[a]	3D CRT[b]	(VMAT-SIB[a])/(3D CRT[b])
Not operated patients excluded (number of patients)	12	6	-
Type of surgery			
Sphincter-sparing surgery	49/63	35/56	1.24 (0.98-1.59)
Resection margin			
Negative	58/63	50/56	1.03 (0.92-1.16)
**Total**	**63 (100%)**	**56 (100%)**	-

[a]VMAT-SIB - group of patients treated with the new radiotherapy technique; [b]3D CRT - group of patients treated with the standard approach; [c]CI – confidence interval

Supplementary Table S3. Comparison of pathohistological tumor characteristics between the two patient groups.

Characteristics	Study group		Ratio (95%CI[c])
	VMAT-SIB[a]	3D CRT[b]	(VMAT-SIB[a])/(3D CRT[b])
Not operated patients excluded (number of patients)	12	6	-
Lymphovascular invasion			
No	48/63	35/56	1.22 (0.95-1.56)
Vascular invasion			
No	54/63	44/56	1.09 (0.92-1.29)
Perineural invasion			
No	57/63	38/56	1.33 (1.09-1.62)
Total	**63 (100%)**	**56 (100%)**	-

[a]VMAT-SIB - group of patients treated with the new radiotherapy technique; [b]3D CRT - group of patients treated with the standard approach; [c]CI – confidence interval

Supplementary Table S4. Comparison of pathohistological stage of disease between the two patient groups.

Characteristics	Study group		Ratio (95%CI[d])
	VMAT-SIB[a]	3D CRT[b]	(VMAT-SIB[a])/(3D CRT[b])
Not operated patients excluded (number of patients)	12	6	-
T in pathological TNM			
T0+T1+T2	41/63	18/56	2.02 (1.33-3.09)
N in pathological TNM			
N0	49/63	32/56	1.36 (1.05-1.77)
Number of positive lymph nodes			
Mean (SD)	0.6 (1.5)	1.3 (2.5)	-0.7 (-1.2 - -0.2)[c]
UICC [e] staging			
pCR[g]+I+II	49/63	32/56	1.36 (1.05-1.77)
Total	**63 (100%)**	**56 (100%)**	-

[a]VMAT-SIB - group of patients treated with the new radiotherapy technique; [b]3D CRT - group of patients treated with the standard approach; [e]UICC, Union for International Cancer Control; [g]pCR – pathologic complete response; [c]difference between two mean values for VMAT-SIB[a] and 3D CRT[b] group; [d]CI – confidence interval

Supplementary Table S5. Comparison of the two patient groups regarding pathological response to treatment.

Characteristics	Study group		Ratio (95%CI[c])
	VMAT-SIB[a]	3D CRT[b]	(VMAT-SIB[a])/(3D CRT[b])
Not operated patients excluded (number of patients)	12	6	-
TRG - groups			
TRG1 (pCR)	13/63	5/56	2.31 (0.88-6.08)
TRG - groups			
TRG1-2	23/63	14/56	1.46 (0.84-2.55)
RCRG - groups			
RCRG1	28/63	12/56	2.07 (1.17-3.68)
Total	**63 (100%)**	**56 (100%)**	**-**

[a]VMAT-SIB - group of patients treated with the new radiotherapy technique; [b]3D CRT - group of patients treated with the standard approach; [c]CI – confidence interval

Supplementary Table S6. Comparison of the two groups of patients in relation to the reduction in disease category and stage.

**Characteristics**	**Study group**		**Ratio (95%CI[c])**
	**VMAT-SIB[a]**	**3D CRT[b]**	**(VMAT-SIB[a])/(3D CRT[b])**
Not operated patients excluded (number of patients)	12	6	-
Reduction of initial T stage			
Yes	45/63	22/56	1.82 (1.27-2.61)
Reduction of initial N stage			
Yes	55/63	44/56	1.11 (0.94-1.31)
Reduction of initial T or N stage			
Yes	58/63	46/56	1.12 (0.97-1.29)
Reduction of initial T and N stages			
Yes	42/63	20/56	1.87 (1.26-2.76)
Downstaging			
Yes	50/63	31/56	1.43 (1.1-1.87)
Total	**63 (100%)**	**56 (100%)**	**-**

[a]VMAT-SIB - group of patients treated with the new radiotherapy technique; [b]3D CRT - group of patients treated with the standard approach; [c]CI – confidence interval
